# Assessment of Maximal Oxygen Uptake (VO2 Max) in Athletes and Nonathletes Assessed in Sports Physiology Laboratory

**DOI:** 10.7759/cureus.61124

**Published:** 2024-05-26

**Authors:** Sujay Srivastava, Shubhi Tamrakar, Naveenkumar Nallathambi, Suryadev A Vrindavanam, Roshan Prasad, Ruchi Kothari

**Affiliations:** 1 Physiology, Mahatma Gandhi Institute of Medical Sciences, Wardha, IND; 2 Physiology, Dr. D. Y. Patil School of Medicine, Mumbai, IND; 3 Internal Medicine, Madras Medical College, Chennai, IND; 4 Medicine and Surgery, Jawaharlal Nehru Medical College, Datta Meghe Institute of Higher Education and Research, Wardha, IND

**Keywords:** cardiorespiratory fitness, maximal oxygen uptake, ergometer, treadmill, athletes

## Abstract

Background: Athletes' physical prowess plays a crucial role in their ability to succeed in any sporting endeavor. Each athlete on the field must have an exceptional aerobic capacity to withstand fierce competition and stringent regulatory guidelines. Maximal oxygen uptake (VO_2_ max) is a quantitative measure of aerobic capacity and is regarded as one of the most reliable indicators of cardiorespiratory and overall physical fitness of an individual by sports physiologists. The study aims to evaluate the VO_2_ max of athletes in comparison with nonathletes during treadmill and lower limb cycle ergometry exercises as assessed in the Sports Physiology Laboratory of a rural medical college. Treadmill exercise and bicycle ergometer exercise are the most common to perform as indoor aerobic exercises to assess one's physical fitness. Both these tests are equally useful in eliciting cardiac and vascular responses, so both these modalities were used to assess aerobic fitness.

Methods: This cross-sectional study, which examined participants aged 17-25, included 30 athletes (cases) and 120 age- and sex-matched controls. The VO_2_ max was evaluated using the Metabolic Module of Lab Chart Software, which was investigated through the PowerLab data acquisition system, AD Instruments (Bella Vista, NSW, Australia).

Results: The mean age of male athletes was 20.51 ± 2.69 years and of female athletes was 20.53 ± 1.62 years. The mean and standard deviation of VO_2_ max on the treadmill for male cases was 52.37 ± 8.78 mL/kg/min and for female cases was 40.96 ± 4.06 mL/kg/min, and on a cycle ergometer for male cases was 45.21 ± 9.43 mL/kg/min and for female cases was 34.32 ± 5.12 mL/kg/min. For the control group, the mean age of control males was 21.2 ± 2.62 years and of control females was 20.36 ± 1.5 years. The mean and standard deviation of VO_2_ max on the treadmill for control males was 33.35 ± 3.77 mL/kg/min and for control females was 25.09 ± 7.07 mL/kg/min, and on the cycle ergometer for control males was 34.17 ± 2.75 mL/kg/min and for control females was 24.15 ± 5.35 mL/kg/min.

Conclusion: This study showed significantly (p < 0.001) higher VO2 max levels in athletes of both genders compared to their age- and sex-matched controls upon exercise on the treadmill and cycle ergometer. This study underscores the significance of better cardiorespiratory fitness in athletes than nonathletes, giving pertinent insights about their aerobic capacity, which is precisely measured and expressed in terms of VO_2_.

## Introduction

The term "athlete" originates from the Greek word "athlos," which means accomplishment and is often linked to extraordinarily high performance. It has traditionally been used in sporting competitions at the current World Championships and the age-old Olympic Games. Compared to the general population of the same age and sex, athletes are commonly viewed as beacons of optimal health. Hence, they have always attracted much attention from the public and the media [1]. It is often interesting to know how physically capable an athlete is. Additionally, a decline in this ability to perform may be a clue to the initial symptom of an illness [2-5]. Athletes are supposed to have stronger cardiorespiratory fitness metrics than nonathletes. Cardiorespiratory fitness is estimated as maximal oxygen uptake, commonly called VO_2 _max. Sports physiologists consider it one of the most reliable indicators of an athlete's physical fitness and cardiorespiratory performance [6]. VO_2_ max predicts improved cardiorespiratory fitness and success in endurance sports. It has been described as the highest volume of oxygen consumed during exercise, beyond which no further increment in oxygen intake is possible with increased exercise intensity [7,8]. It is measured in liters of O_2_/min or milliliters of O_2_/kg of body weight/minute [8]. The international benchmark for physical ability is the VO_2_ max [9]. Oxygen uptake must reach a plateau to quantify maximum oxygen uptake, which describes the efficient integration of the brain, cardiopulmonary, and metabolic systems. Incremental exercise does not always result in a plateau, so the VO_2 _peak obtained during a maximum-effort incremental test will likely be a reliable indicator of VO_2 _max [10].

Over the past four decades, maximal aerobic capacity has emerged as a robust predictor of negative health consequences and overall mortality risk. To combat adverse outcomes, VO_2_ max can be enhanced by doing exercise across different intensity levels [11]. The results from the study by Montero and Díaz-Cañestro regarding the effects of physical activity on the age-related decline in VO_2 _max suggested that endurance exercise training reduces the rate of decline in maximal heart rate (HR) and age-related decrease in VO_2_ max [11].

Although many researchers [9-11] have reported the efficacy of high-intensity training in enhancing VO_2_ max, these studies have often recruited small samples. Bute et al. [12], in their study done exclusively on 50 females between the age group of 18-22 years, classified 25 athletes as the study group and 25 nonathletes as controls. They determined VO_2_ max through the Queen’s College step test and compared them. In a study by Gupta et al. [13] conducted in a tertiary-care hospital on 100 male subjects who underwent regular exercise training for one year, increased VO_2_ max, physical fitness index, and reduced resting pulse rate and blood pressure were reported.

While some investigators [14-16] have highlighted the significance of both high- and low-intensity exercises for raising VO₂ max, it is surprising that there is sparse literature suggesting what the ideal workout intensity might be, despite the clear significance of identifying the optimal exercise training for an athlete.

Further, it is evident from the literature review that most of the VO₂ max studies are either on male athletes or on female athletes. Hardly any study has been undertaken in both sexes together, especially in the Indian subcontinent, so we intended to assess VO₂ max levels in athletes of both genders and compare these levels in nonathlete individuals with a sedentary lifestyle. This study may contribute to the knowledge transfer of the next generation regarding the importance of physical fitness in preventing chronic diseases associated with lifestyle choices. For adults currently in good health, exercise is a potentially significant preventative strategy to promote cardiovascular health and diminish overall mortality from diseases. Consequently, these benefits of exercise highlight its importance for sustaining the overall physiological, psychological, and emotional well-being of athletes and nonathletes. The study aimed to evaluate the VO₂ max of athletes upon exercise on the treadmill and cycle ergometer as assessed in the Sports Physiology Laboratory of a rural medical college.

This study was previously presented as an oral presentation in Colombo, Sri Lanka, at the International Medical Undergraduate Conference on February 7, 2019, where it was conferred the second best research paper award.

## Materials and methods

Study design

It was a cross-sectional study. The Strengthening the Reporting of Observational Studies in Epidemiology guidelines were followed for reporting and preparing the manuscript.

Study setting

The study was conducted in a rural medical college in central India's Sports Physiology Laboratory by the Department of Physiology.

Study participants

A total of 30 athletes who had participated in sports like swimming, basketball, badminton, football, handball, or other athletic activities like brisk walking, gym training, running, jogging, or cycling were regarded eligible as cases for the study in the age range of 17-25 years. The definition of an athlete given by the American Heart Association was considered for the recruitment of athletes in the present study [17]. The controls comprised 120 age-matched (age of the athlete ±5 years) and sex-matched sedentary individuals who abstained from exercise or did not play any outdoor games as was detailed in the history of their physical activity in the previous month.

A signed written informed consent was obtained from all study participants. Prior approval from the Institutional Ethics Committee was ensured before the beginning of the study, and the corresponding ethics approval number is MGIMS/IEC/PHY/127/1/2017.

Sample size and sampling technique

The sample size was 150, including 30 cases (athletes) and 120 controls (nonathletes). All 150 subjects were recruited for the study using the convenient sampling technique. This sample size was estimated using the OpenEpi 3.01 statistical software (Centers for Disease Control and Prevention, Atlanta, GA) with assumptions such as a 95% confidence level, 80% power of the study, and a 1:4 case-to-control ratio to make the study's results more robust, credible, and authentic [18].

Inclusion Criteria

Subjects who gave written informed consent and had no underlying medical or psychiatric illness, as detailed in their history, general physical examination, and clinical examination, were included in the study. They were not taking any medications affecting the cardiorespiratory responses.

Exclusion Criteria

Tobacco addicts, alcoholics, smokers, and people with a known history of diabetes and hypertension, chronic illnesses, cardiovascular or respiratory diseases, any major systemic or psychiatric illness, or physical disability were not included in the study.

Data sources and measurement of variables

All subjects were instructed to reach the Sports Physiology Laboratory relaxed and fully hydrated. All recordings were made a minimum of two hours after food intake. It was made sure that participants refrained from tea, alcohol, caffeine, tobacco, alcohol, intense exercise, and smoking/nicotine for at least 12 hours before an experiment [9]. The subjects were required to fill in a proforma before proceeding to testing. History was recorded, followed by anthropometry and clinical examination. The resting pulse, blood pressure, respiratory rate, and VO_2_ max were measured. The height was measured in meters and weight in kilograms as per standard procedures.

Body mass index (BMI) and body surface area (BSA) were evaluated as per standard reference [18] formulas:

BMI = Weight/Height^2^

BSA = 0.20247 × Height^0.725^ × Weight^0.425^

PowerLab 8/35 System from AD Instruments (Bella Vista, NSW, Australia) and LabChart Pro software with its Metabolic Module were used to determine the cardiorespiratory measurements. This module included a Bio Amp, spirometer and flow head, gas analyzer, gas mixing chamber, thermistor pod, and accessories. It recorded the inspired or expired airflow from a pneumotach (attached to the gas mixing chamber) and CO_2_ and O_2_ concentrations from the gas analyzer by the expired air in the gas mixing chamber. The simultaneous measurement of respiratory gas concentrations and airflow helped calculate the metabolic values displayed on the computer screen. It automatically calculated the VO_2_, i.e., oxygen consumption in L/min representing absolute oxygen intake and in mL/kg/min indicating relative oxygen intake, VCO_2_ representing carbon dioxide production (L/min), and respiratory exchange ratio (RER).

The RER is the proportion of oxygen used to carbon dioxide (CO_2_) produced during metabolism. RER calculation is frequently performed with exercise tests like VO_2 _max estimation and can be used as a sign that participants are nearing their physical limits and cardiorespiratory system. The peak value of VO_2_ obtained during an incremental exercise test to volitional exhaustion was used in this study to define aerobic capacity. A supplementary endpoint criterion for a VO_2_ peak was frequently utilized when the RER was greater than or equal to 1.15.

After becoming comfortable with the lab and procedures, the participants performed an incremental ramp exercise test to the limit of tolerance on a motorized treadmill (Aerofit AF 101, Nityasach Fitness Pvt Ltd, Mumbai, India) before using a motorized bicycle ergometer (Aerofit AF 176U, Nityasach Fitness Pvt Ltd, Mumbai, India) during a five-to-eight-minute increasing-speed run to fatigue.

The participants commenced with a five-minute warm-up at 25 watts before undergoing maximal testing to measure VO₂ max. Throughout the estimation, the pedal rate was kept constant at 60 rpm. The investigation began with unloaded cycling at 60 cycles per minute for two minutes. Subsequently, the workload was incremented by 29 watts (equivalent to 0.5 kg) every two minutes until it reached 118 watts. Following this, the power output was raised by 15 watts (0.25 kg) until the participants voluntarily stopped or a decrease in the pedal rate of five cycles per minute was observed [19].

For the treadmill exercise, speeds were selected to gradually progress in increments from moderate to maximal intensity with zero inclination. The speed was increased from 0 to 4 km/hour in the first minute and subsequently increased by 1 km/hour every minute until the subject was exhausted and wished to stop [9]. At least two of the following requirements had to be satisfied to be regarded as a legitimate maximal test, namely a peak or a plateau in oxygen uptake, O₂ consumption (VO₂), RER ≥1.15, and maximal HR age-predicted maximum [20].

A wrist-worn pulse oximeter was utilized to track HR consistently throughout exercise sessions, providing immediate HR measurements in real time.

Statistical analysis of data

Means and standard deviations were calculated to describe participants’ characteristics by sex. Statistical Software IBM SPSS Statistics for Windows, IBM Corp, Version 28.0 (Armonk, NY) was used to conduct the statistical analysis, which included the unpaired Student’s t-test. Analysis of variance was used to test differences between the study groups for anthropometric measures and age. Statistical significance was defined as a p value of less than 0.05.

## Results

A total of 30 cases (athletes) and 120 age-matched healthy young controls were investigated in the Sports Physiology laboratory to determine the cardiorespiratory parameters, particularly VO_2_, i.e., oxygen consumption and RER (respiratory gas ratio). The proportion of males and females was the same in both study groups: 53.33% were males (64 of 120) and 46.66% were females (56 of 120). Among the cases, 16 were males, and 14 were females.

There was no statistically significant difference in the mean age, height, weight, BMI, and BSA of the two study groups, as depicted in Tables 1, 2. However, the HR was lower in cases than in controls.

**Table 1:** Descriptive statistics of males BMI: body mass index; BSA: body surface area; SD: standard deviation; S: significant; NS: not significant All the p values reported are based on the Student’s t-test between the case and control groups

S. no.	Parameters	Control males (n = 64), mean ± SD	Case males (n = 16), mean ± SD	P value
1	Age (years)	21.2 ± 2.62	20.51 ± 2.69	0.351 (NS)
2	Height (cm)	171.18 ± 5.27	173.05 ± 6.65	0.232 (NS)
3	Weight (kg)	68 ± 9.5	66.71 ± 10.93	0.638 (NS)
4	Heart rate (beats/min)	82 ± 7	75 ± 8	<0.001 (S)
5	BMI (kg/m^2^)	23.3 ± 3.86	22.31 ± 3.59	0.355 (NS)
6	BSA (m^2^)	1.8 ± 0.10	1.79 ± 0.14	0.743 (NS)

**Table 2:** Descriptive statistics of females BMI: body mass index; BSA: body surface area; SD: standard deviation; S: significant; NS: not significant All the p values reported are based on the Student’s t-test between the case and control groups

S. no.	Parameters	Control females (n = 56), mean ± SD	Case females (n = 14), mean ± SD	P value
1	Age (years)	20.36 ± 1.5	20.53 ± 1.62	0.710 (NS)
2	Height (cm)	161.95 ± 4.24	162.29 ± 5.22	0.798 (NS)
3	Weight (kg)	59.64 ± 8.05	56.65 ± 10.22	0.243 (NS)
4	Heart rate (beats/min)	85 ± 8	72 ± 4	<0.001 (S)
5	BMI (kg/m^2^)	22.75 ± 3.11	21.45 ± 3.34	0.172 (NS)
6	BSA (m^2^)	1.63 ± 0.10	1.59 ± 0.14	0.222 (NS)

In Table 3, the mean and standard deviation of values of O_2_ consumption and RER for males (controls and cases) are depicted for both pre- and postphase of exercise on a treadmill and a cycle ergometer. There was no statistically significant difference in the VO_2 _and RER levels before exercise, but there was a statistically significant difference in the VO_2_ and RER levels between the controls and cases after the exercise.

**Table 3:** Oxygen consumption and respiratory exchange ratio in males VO2: oxygen consumption; RER: respiratory exchange ratio; SD: standard deviation; S: significant; NS: not significant All the p values reported are based on the Student’s t-test between the case and control groups

S. no.	Parameters	Control males (n = 64), mean ± SD	Case males (n = 16), mean ± SD	P value
Before exercise				
1	VO_2 _(mL/kg/min) (treadmill)	4.21 ± 1.63	5.13 ± 2.66	0.082 (NS)
2	VO_2 _(mL/kg/min) (ergometer)	6.72 ± 3.09	7.34 ± 2.03	0.332 (NS)
3	VO_2 _(L/min) (treadmill)	0.30 ± 0.13	0.35 ± 0.14	0.179 (NS)
4	VO_2 _(L/min) (ergometer)	0.49 ± 0.28	0.55 ± 0.41	0.489 (NS)
5	RER	0.73 ± 0.23	0.78 ± 0.15	0.412 (NS)
After exercise				
6	VO_2 _(mL/kg/min) (treadmill)	5.64 ± 2.04	8.6 ± 2.73	0.0001 (S)
7	VO_2 _(mL/kg/min) (ergometer)	6.19 ± 2.13	8.44 ± 3.16	0.001 (S)
8	VO_2 _(L/min) (treadmill)	0.52 ± 0.41	0.8 ± 0.16	0.009 (S)
9	VO_2 _(L/min) (ergometer)	0.42 ± 0.23	0.62 ± 0.38	0.008 (S)
10	RER	0.75 ± 0.15	0.9 ± 0.2	0.001 (S)

Table 4 shows the mean and standard deviation of O_2_ consumption and RER for females (controls and cases) for both pre- and postphase of exercise on a treadmill and a cycle ergometer. There was no statistically significant difference in the VO2 and RER levels prior to exercise, but the postexercise VO_2_ and RER levels between the controls and cases were statistically different.

**Table 4:** Oxygen consumption and respiratory exchange ratio in females VO2: oxygen consumption; RER: respiratory exchange ratio; SD: standard deviation; S: significant; NS: not significant All the p values reported are based on the Student’s t-test between the case and control groups

S. no.	Parameters	Control females (n = 56), mean ± SD	Case females (n = 14), mean ± SD	P value
Before exercise				
1	VO_2 _(mL/kg/min) (treadmill)	3.4 ± 1.71	4.05 ± 3.38	0.311 (NS)
2	VO_2 _(mL/kg/min) (ergometer)	5.03 ± 3.48	6.5 ± 2.56	0.143 (NS)
3	VO_2 _(L/min) (treadmill)	0.22 ± 0.12	0.29 ± 0.17	0.078 (NS)
4	VO_2 _(L/min) (ergometer)	0.29 ± 0.18	0.32 ± 0.15	0.567 (NS)
5	RER	0.45 ± 0.12	0.5 ± 0.22	0.251 (NS)
After exercise				
6	VO_2 _(mL/kg/min) (treadmill)	4.83 ± 2.4	7.40 ± 3.37	0.001 (S)
7	VO_2_(mL/kg/min) (ergometer)	5.22 ± 1.96	6.95 ± 3.01	0.010 (S)
8	VO_2 _(L/min) (treadmill)	0.31 ± 0.17	0.44 ± 0.11	0.008 (S)
9	VO_2 _(L/min) (ergometer)	0.35 ± 0.14	0.51 ± 0.11	0.0002 (S)
10	RER	0.6 ± 0.22	0.85 ± 0.15	0.0002 (S)

Tables 5, 6 represent the VO_2_ max and RER max data on treadmill exercise for males and females (controls and cases). RER max mentioned in this context is the value of RER achieved at the peak VO_2 _max level. VO_2 _max was significantly higher in the cases than in the control group for both genders. Maximal RER was marginally higher in cases than in the controls in both males and females. According to the data analysis, there was a statistical difference (p < 0.001) in all the variables (VO_2 _max, RER max) between the controls and cases for both males and females.

**Table 5:** Treadmill maximal data for males VO_2_: oxygen consumption; RER: respiratory exchange ratio; SD: standard deviation; S: significant All the p values reported are based on the Student’s t-test between case and control groups

Variable	Control males (n = 64), mean ± SD	Case males (n = 16), mean ± SD	P value
VO_2 _max (mL/kg/min)	33.35 ± 3.77	52.37 ± 8.78	<0.001 (S)
VO_2 _max (L/min)	2.28 ± 0.50	3.41 ± 0.57	<0.001 (S)
RER max	0.97 ± 0.06	1.04 ± 0.06	0.006 (S)

**Table 6:** Treadmill maximal data for females VO_2_: oxygen consumption; RER: respiratory exchange ratio; SD: standard deviation; S: significant All the p values reported are based on the Student’s t-test between the case and control groups

Variable	Control females (n = 56), mean ± SD	Case females (n = 14), mean ± SD	P value
VO_2 _max (mL/kg/min)	25.09 ± 7.07	40.96 ± 4.06	<0.001 (S)
VO_2 _max (L/min)	1.59 ± 0.51	2.29 ± 0.29	<0.001 (S)
RER max	0.98 ± 0.07	1.03 ± 0.06	0.016 (S)

Tables 7, 8 represent the VO_2 _max data on the cycle ergometer for both males and females (controls and cases). VO_2 _max was significantly higher in the cases than in the control group in both males and females. Maximal RER was marginally higher in cases than in the controls for both males and females. There was a statistical difference (p < 0.001) in the values of VO_2_ max between controls and cases in both genders. However, there was no statistical difference in the RER max values between the controls and cases for both males and females.

**Table 7:** Ergometer maximal data for males VO_2_: oxygen consumption; RER: respiratory exchange ratio; SD: standard deviation; S: significant; NS: not significant All the p values reported are based on the Student’s t-test between the case and control groups

Variable	Control males (n = 64), mean ± SD	Case males (n = 16), mean ± SD	P value
VO_2 _max (mL/kg/min)	34.17 ± 2.75	45.21 ± 9.43	<0.001 (S)
VO_2_ max (L/min)	2.24 ± 0.43	2.98 ± 0.56	<0.001 (S)
RER max	0.99 ± 0.08	1.0 ± 0.09	0.663 (NS)

**Table 8:** Ergometer maximal data for females VO_2_: oxygen consumption; RER: respiratory exchange ratio; SD: standard deviation; S: significant; NS: not significant All the p values reported are based on the Student’s t-test between the case and control groups

Variable	Control females (n = 56), mean ± SD	Case females (n = 14), mean ± SD	P value
VO_2 _max (mL/kg/min)	24.15 ± 5.35	34.32 ± 5.12	<0.001 (S)
VO_2 _max (L/min)	1.50 ± 0.44	1.92 ± 0.37	<0.001 (S)
RER max	0.98 ± 0.07	0.99 ± 0.06	0.625 (NS)

## Discussion

Aerobic capacity is a crucial component of sporting success and affects an athlete's performance on the field. It has been argued that even though insufficient exercise will grant the beneficial adaptations needed for health and disease prevention, too much can cause harm. VO_2 _max is a quantitative measure of an athlete's ability to transport oxygen and indicates the overall efficiency of cardiopulmonary function, serving as a reliable measure of endurance in sports [21]. Enhancing aerobic capacity by choosing an effective training regimen based on their VO_2_ max remains critical for athletes aiming to boost their performance. For the current study, the athletes and nonathletes were subjected to exercise on a treadmill and a cycle ergometer to determine the VO_2_ max in them. The parameters evaluated in study subjects were pre- and postexercise VO_2_, RER, VO_2_ max in mL/kg/min, VO_2_ max in L/min, and RER max.

According to concepts of sports physiology, VO_2 _max refers to a person's capacity to boost metabolic functions in response to higher physical demands [22]. This occurs due to converting chemical energy to mechanical energy [23]. Age, gender, genetics, body composition, training condition, and exercise modality are only a few variables affecting VO_2_ max. Physical exercise training raises stroke volume, increasing VO_2_ max by 50%. The remaining 50% of the rise results from increased oxygen extraction by exercising musculature, as indicated by their raised arteriovenous difference. The intensive bouts of aerobic exercise can cause all muscles to expand significantly. The density of capillaries in skeletal muscles is also increased by exercise-increased vascularization, which results from this improved ability to irrigate the muscles with blood [24]. The number of mitochondria increases, as does their ability to produce adenosine triphosphate by oxidative phosphorylation in an aerobic fashion [21]. High VO_2_ max can be ascribed to a particular type of activity in athletes. More energy and greater athletic performance result from high VO_2_ max, which reduces metabolic demands on the cardiac muscle, spares the heart from undue exertion, and helps increase cardiac output when required.

Our study's athletic group had lower resting pulse rates, which suggests that aerobic exercise causes adaptive changes in the cardiovascular system. These include reduced resting pulse rate and greater resting stroke volume due to heart muscle hypertrophy. These two elements cause athletes' VO_2 _max to rise. As it has been known, exercise improves maximal cardiac output and maximal oxygen extraction by tissues, which are crucial for VO_2_ max [25].

Bute et al. reported a mean VO_2_ max of 39.35 ± 2.78 mL/kg/min in female athletes of their study [12]. Females who lead inactive lifestyles had an average VO_2_ max of 25.08 ± 3.48 mL/kg/min. In our study, VO_2_ max values in athletic females were 40.96 ± 4.06, whereas in control females, it was 25.09 ± 7.07 mL/kg/min.

According to the study by Gupta et al. [13], the VO_2_ max value in male athletes was 61.2 ± 6.2 mL/kg/min, while in controls, it was 43.2 ± 4 mL/kg/min. However, in our study, the VO_2_ max value in athletic males was 52.37 ± 8.78 mL/kg/min, and in control males, it was 33.35 ± 3.77 mL/kg/min for treadmill exercise.

Hawkins et al. [23] in their study obtained VO_2_ max values of 4.3 ± 0.1 L/min and 58.7 ± 1.7 mL/kg/min and RER max value of 1.15 ± 0.2 for male athletes after exercise. In female athletes, VO_2_ max was 2.7 ± 0.1 L/min and 48.7 ± 1.6 mL/kg/min and RER max was 1.09 ± 0.2. The RER enables the differentiation of young male athletes for aerobic performance. On the other hand, in our study, in athletic males, we found VO_2_ max values as 3.41 ± 0.57 L/min and 52.37 ± 8.78 mL/kg/min and RER max as 1.04 ± 0.06 on treadmill exercise. In athletic females, VO_2_ max values were recorded as 2.29 ± 0.29 L/min and 40.96 ± 4.06 mL/kg/min, and RER max value was 1.03 ± 0.06 on treadmill exercise.

Our observations corroborated well with the study of VO_2_ max conducted by Laskowski et al. [24]. The highest VO_2 _max of their athletes on cycle ergometer was 3.2 L/min and 59.42 mL/kg/min. In comparison, in our study, the VO_2_ max values of athletic males on an ergometer were 2.98 ± 0.56 L/min and 45.21 ± 9.43 mL/kg/min. VO_2_ max values of athletic females on the ergometer were 1.92 ± 0.37 L/min and 34.32 ± 5.12 mL/kg/min.

In agreement with Abiodun et al. [26], who reported the peak oxygen uptake was significantly higher on the treadmills than on the ergometers, our results have also shown similar findings. Treadmill running generally entails greater intensity than stationary cycling, leading to increased cardiovascular stress. Moreover, central neural control mechanisms react to the physiological adjustment, affecting HR and blood pressure regulation during exercise [21].

We recommend that the athletes should employ each test modality independently for a comprehensive fitness evaluation and subsequent training strategies. In the realm of sports science, the statistics of maximal data on treadmills and ergometers can be optimally utilized to gauge the extent of cardiovascular function decline and strategize rehabilitation apart from serving to evaluate athletes' fitness levels.

Limitations

While the study offers valuable insights into aerobic capacity in athletes, it is important to acknowledge a few limitations when interpreting the findings. These limitations encompass concerns regarding sample size, discrepancies in exercise protocols, and the absence of long-term analysis. Furthermore, environmental factors and the accuracy of measurement equipment could have impacted the results. Due to time constraints associated with a short-term studentship research project, the sample size was limited. Additionally, the age range had to be confined to align with the target population of athletes and controls.

## Conclusions

When comparing individuals or groups, observing people over time, or using various exercise modalities, it is physiologically important to obtain accurate and valid VO_2 _max data. Its measurement (usually done on a treadmill or cycle ergometer) calls for oxygen intake to reach a point where additional increases in work rate do not result in further increases in VO_2_. In the current study, athletes of both sexes had significantly greater VO_2_ max values than age- and sex-matched controls. A better understanding of the link between VO_2 _and exercise can help us determine the appropriate activity level for different players participating in different sports.
